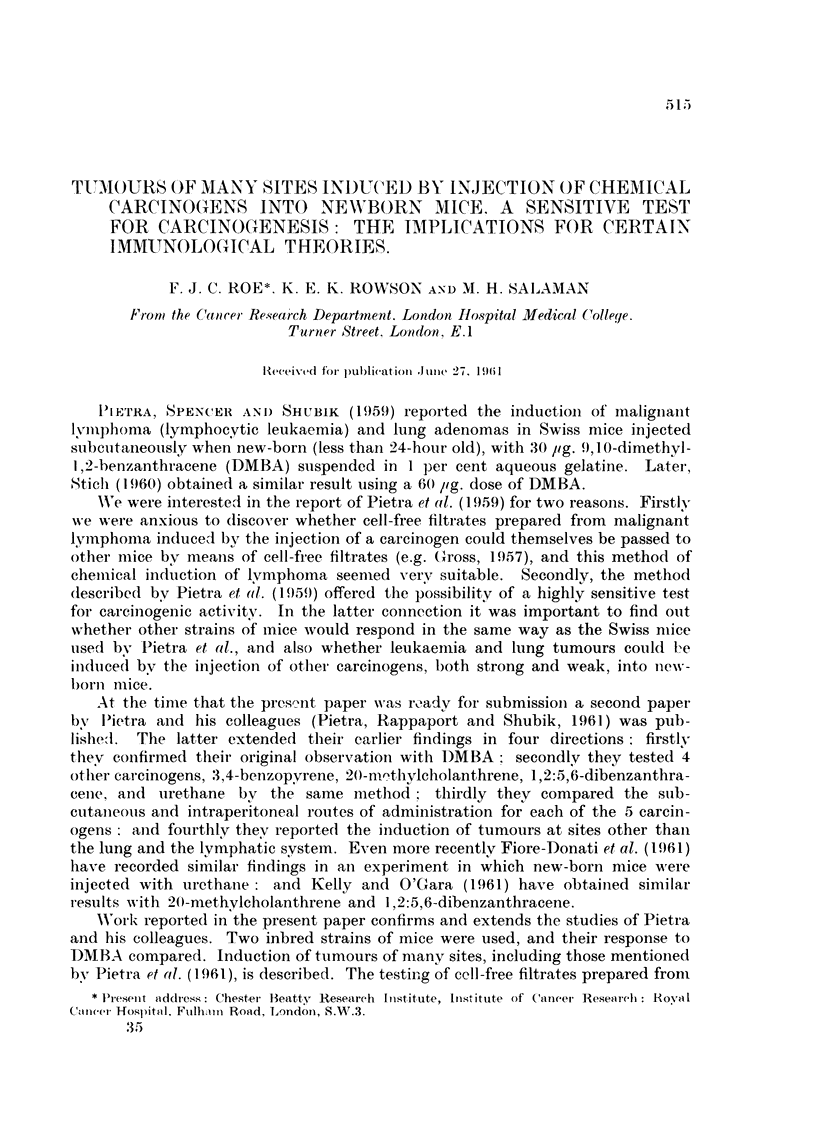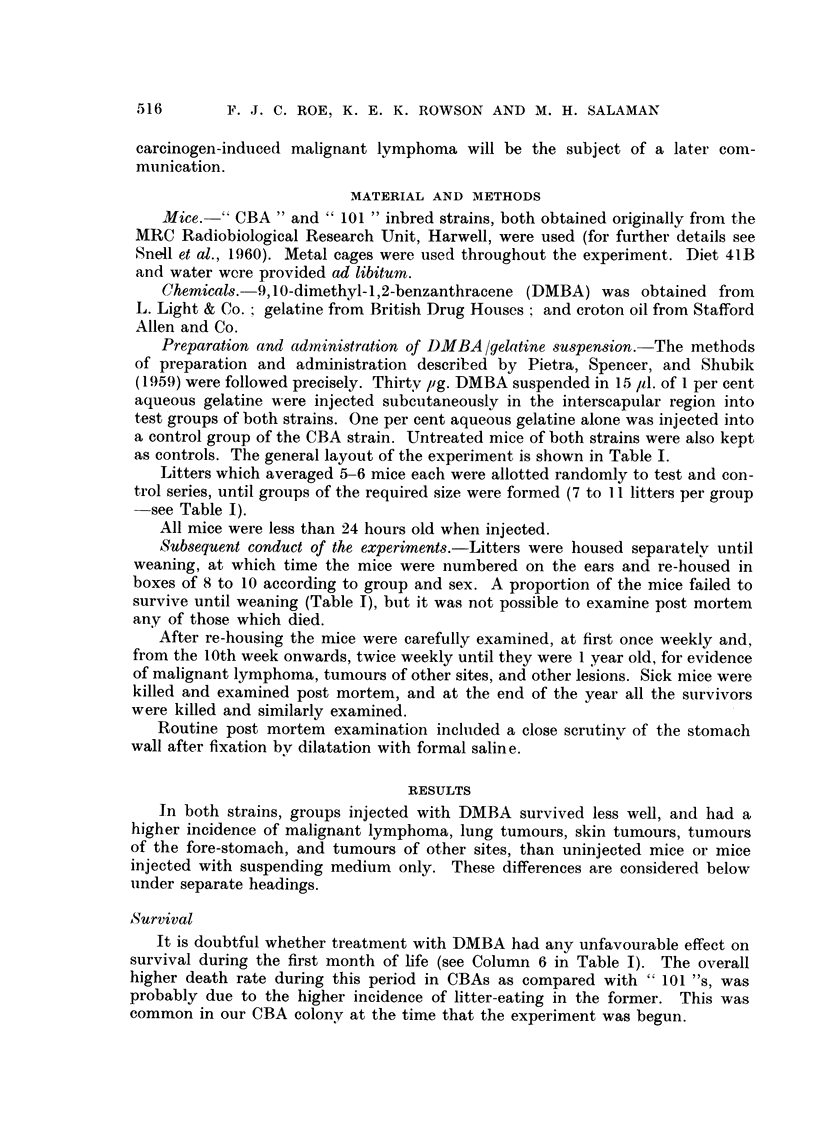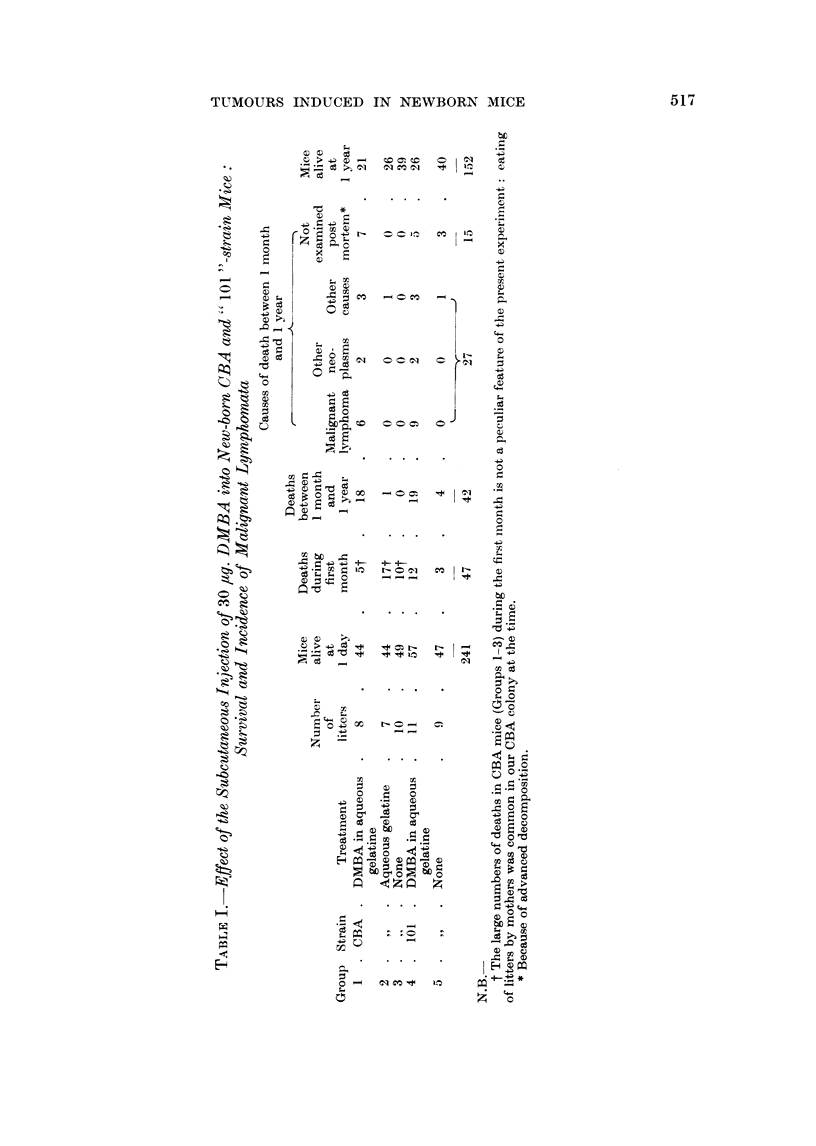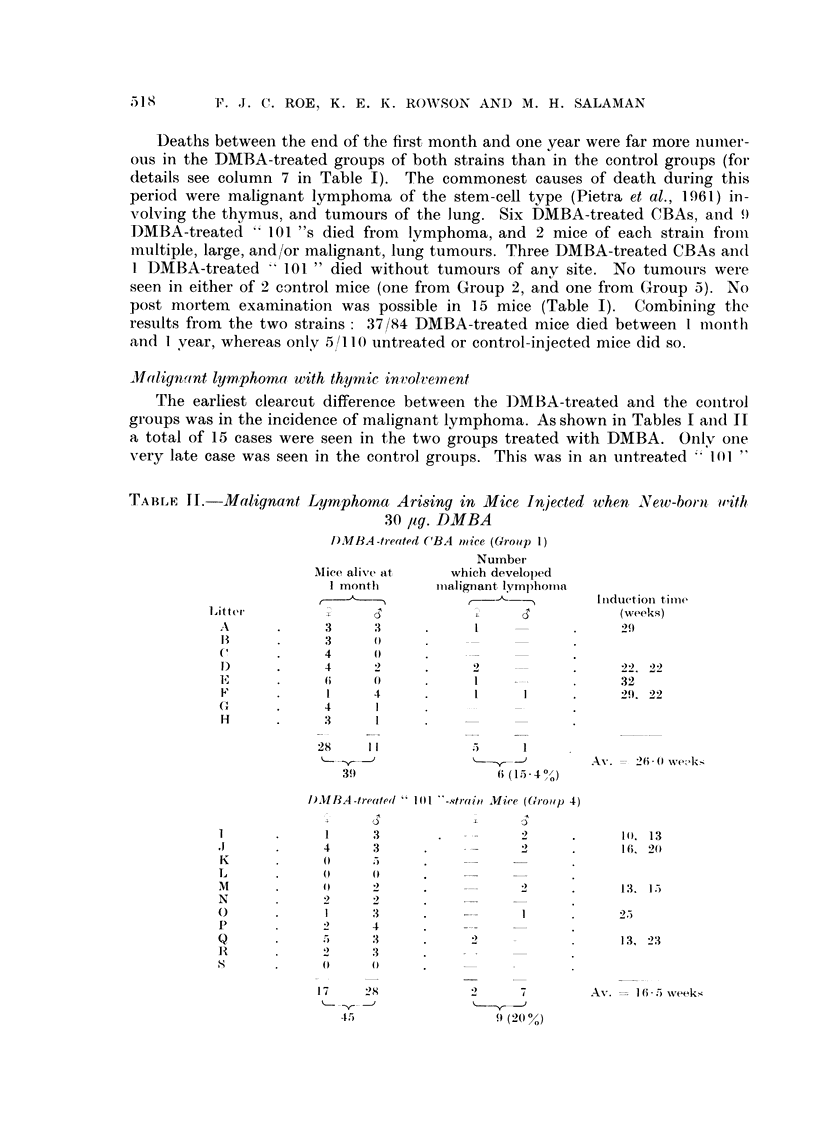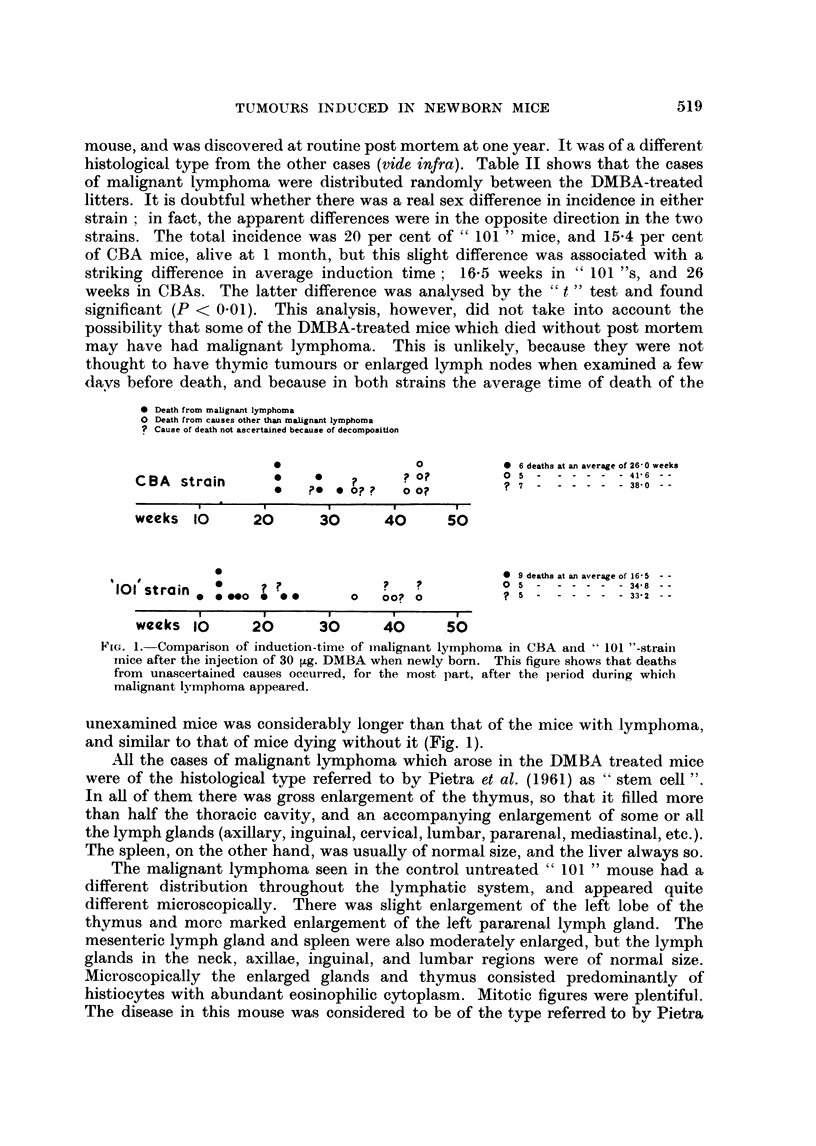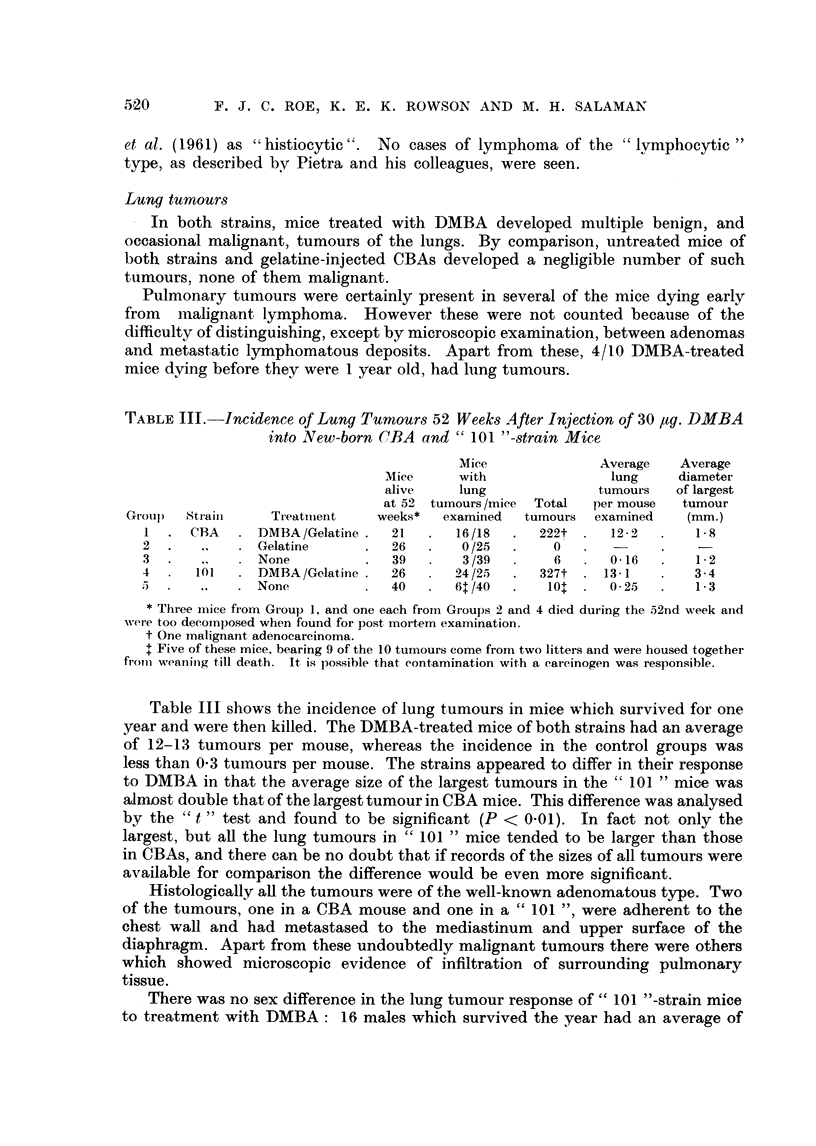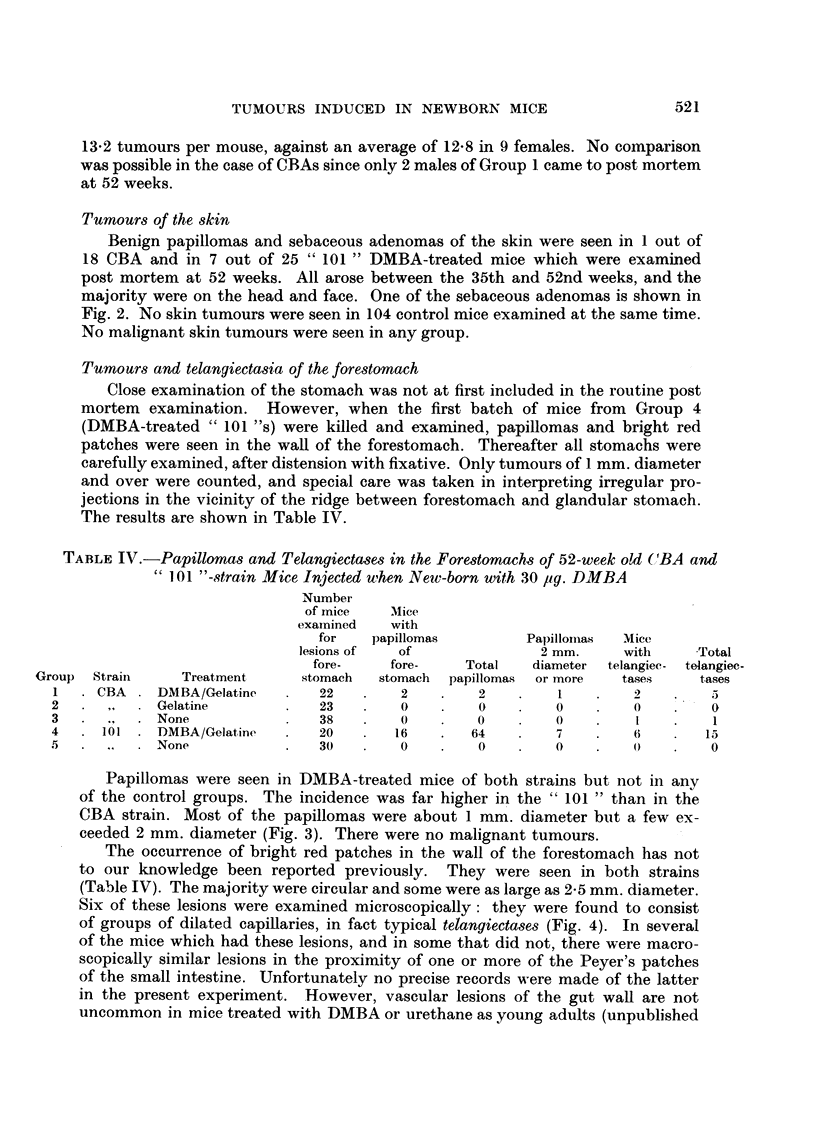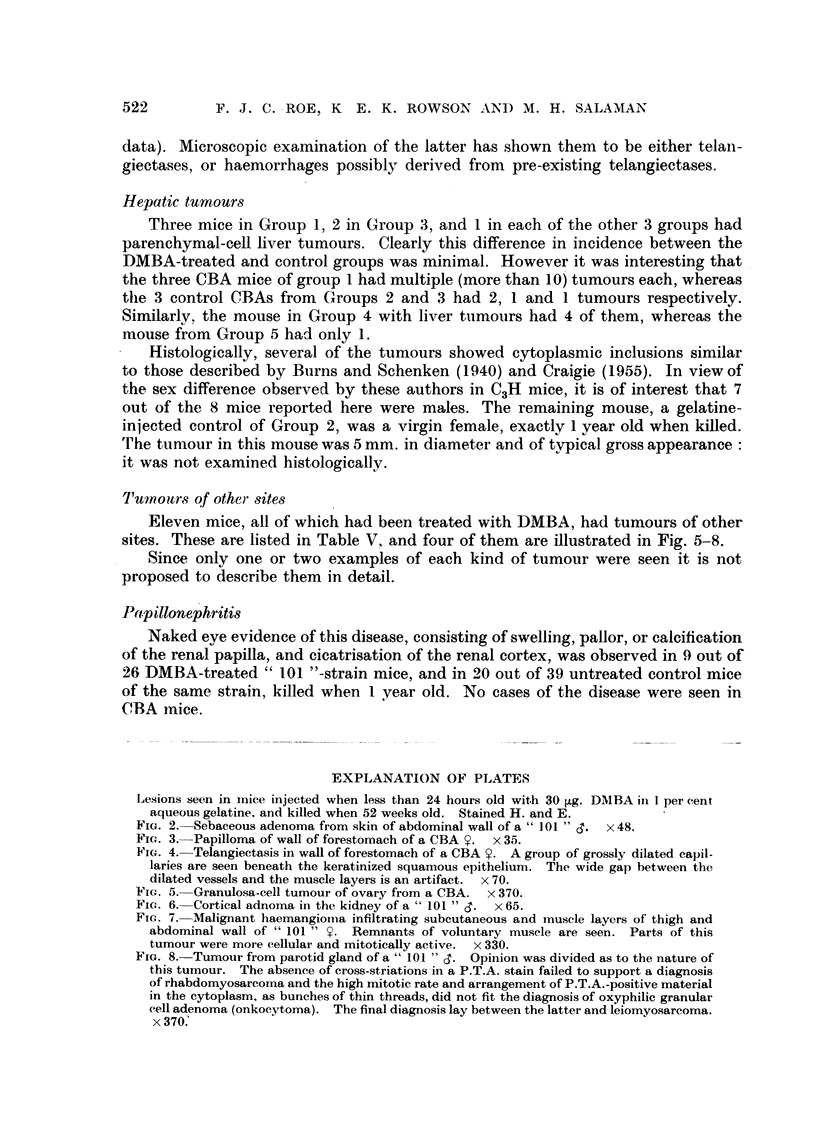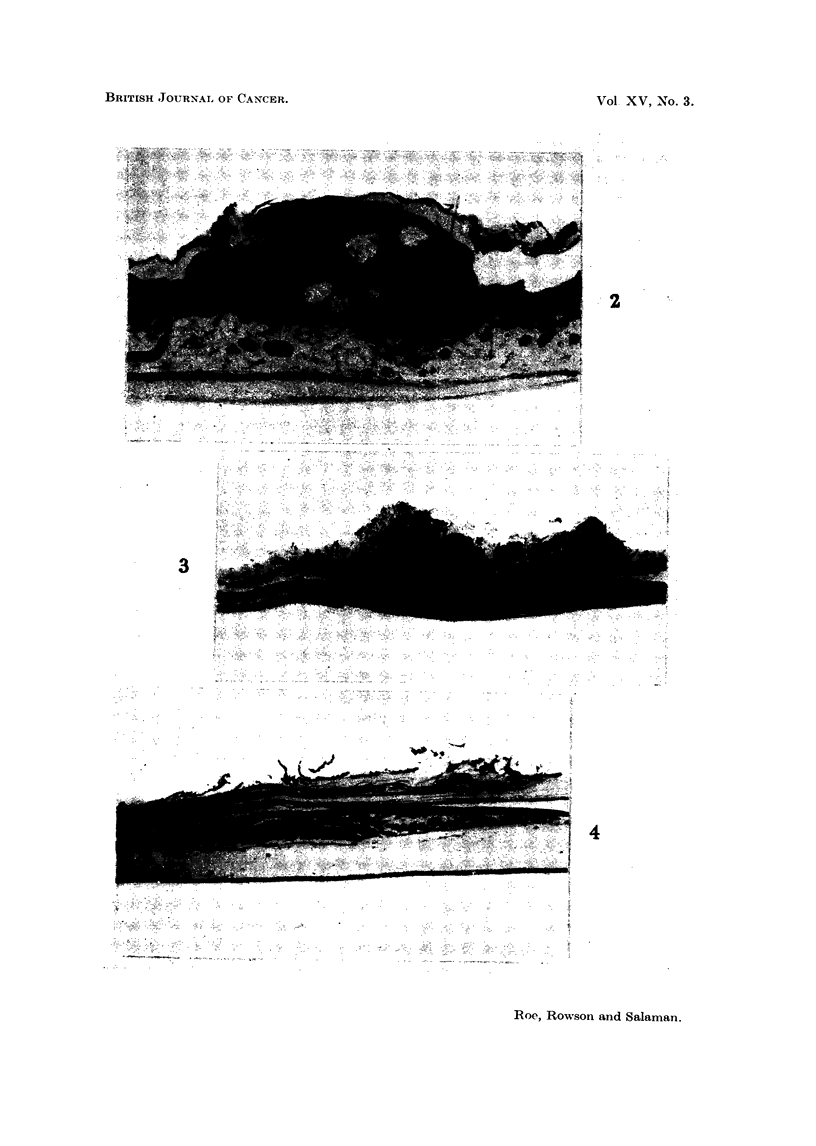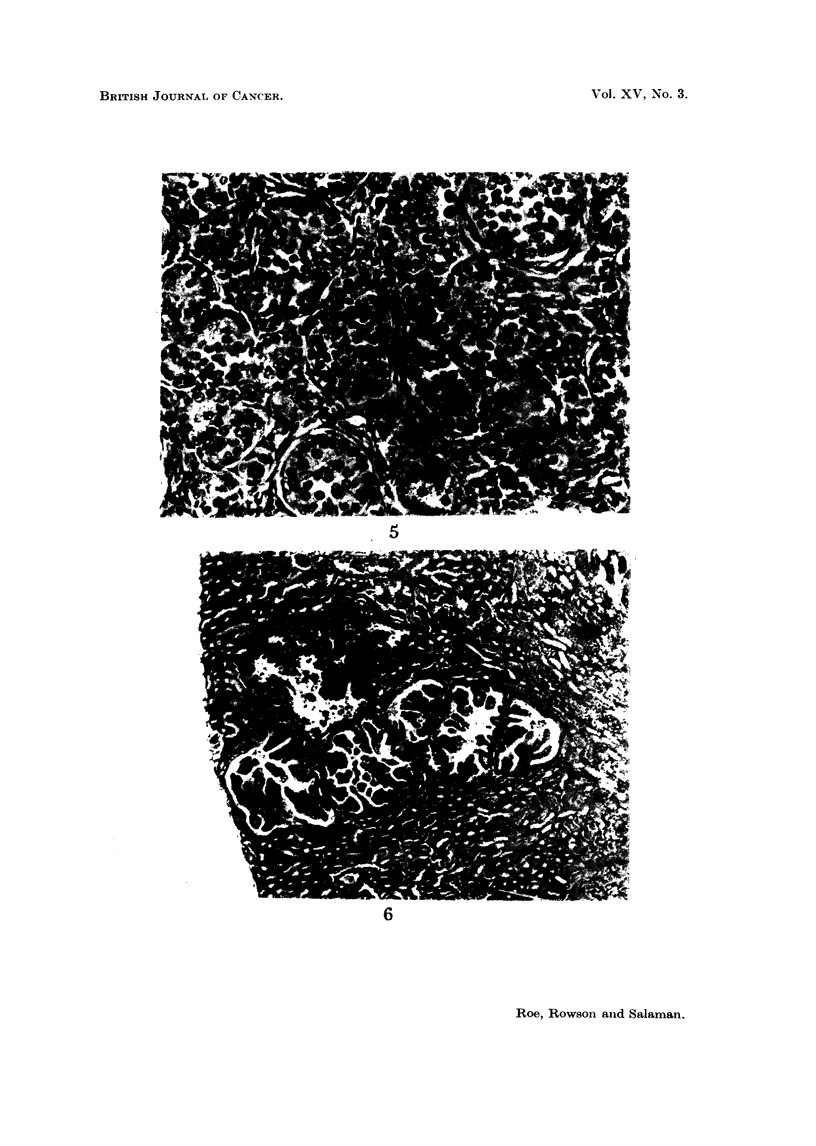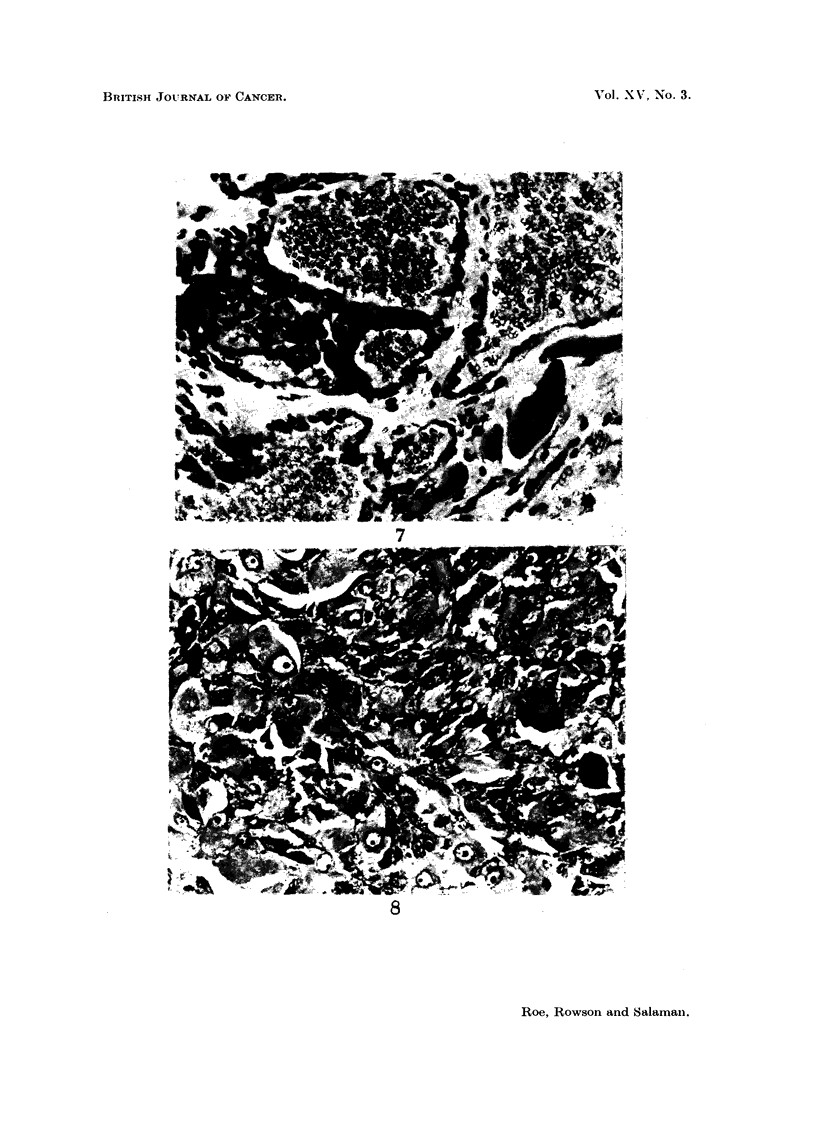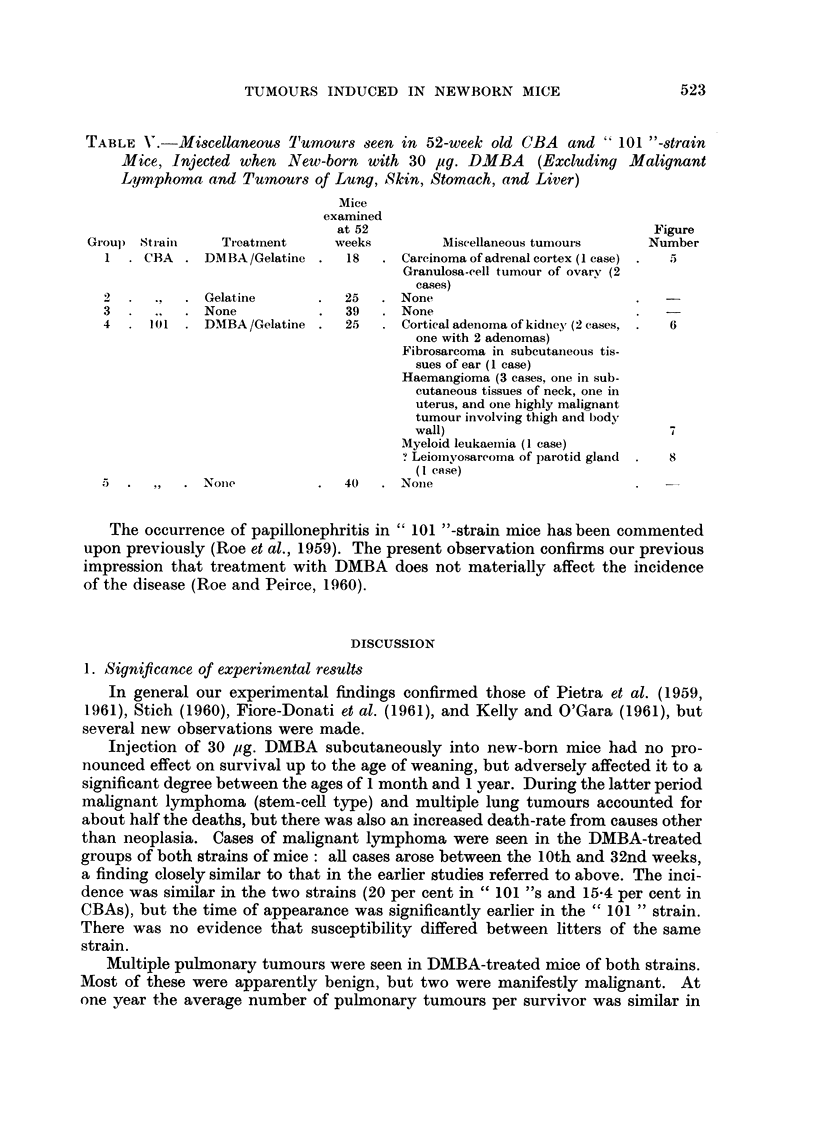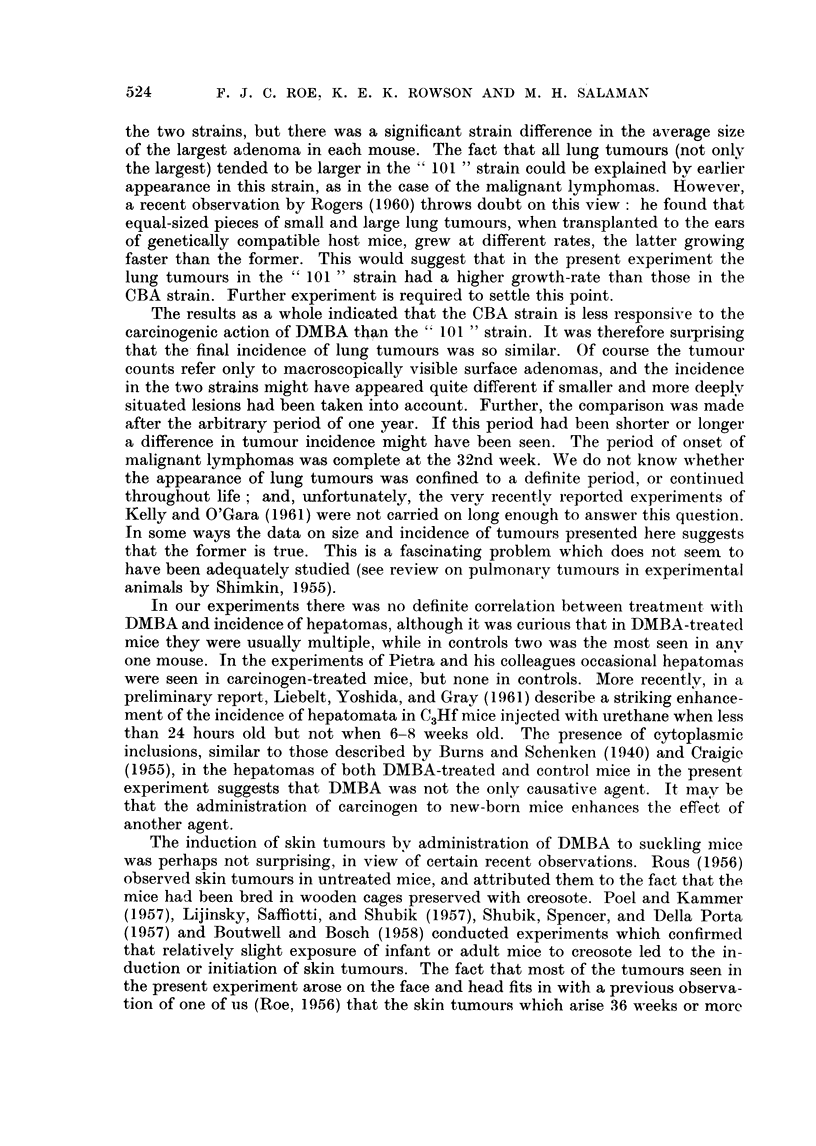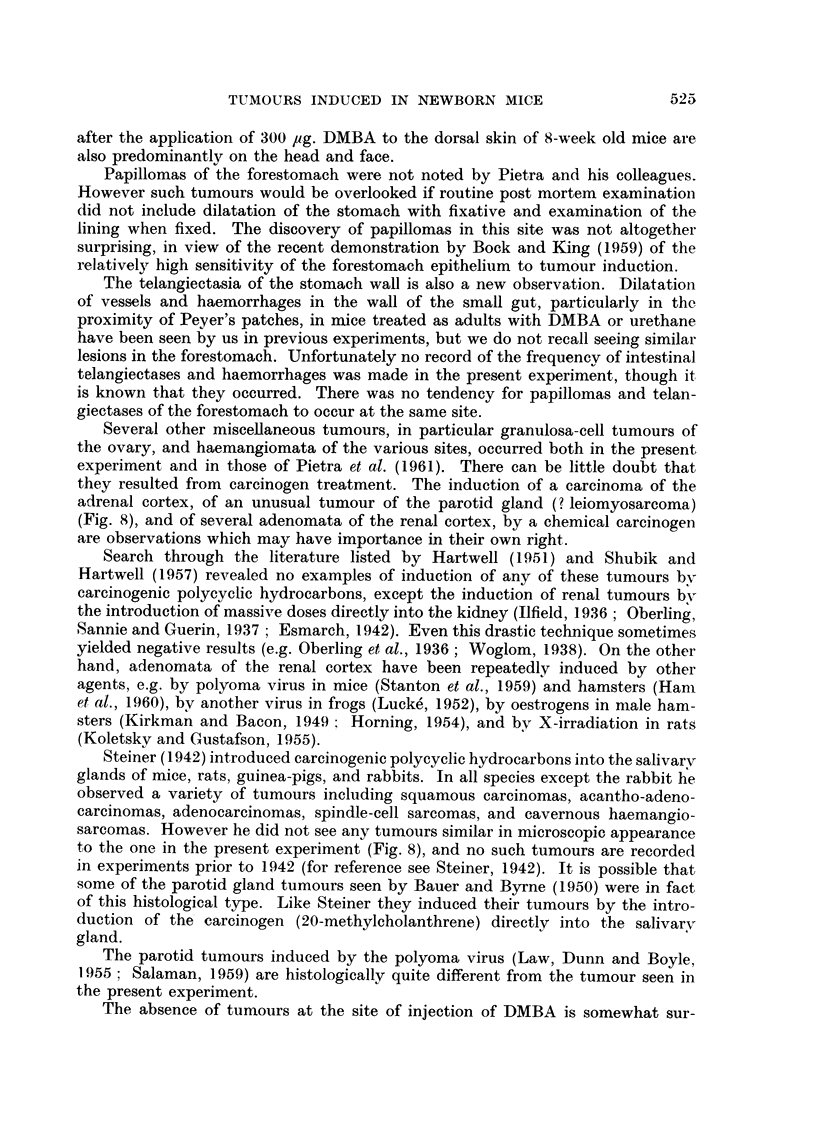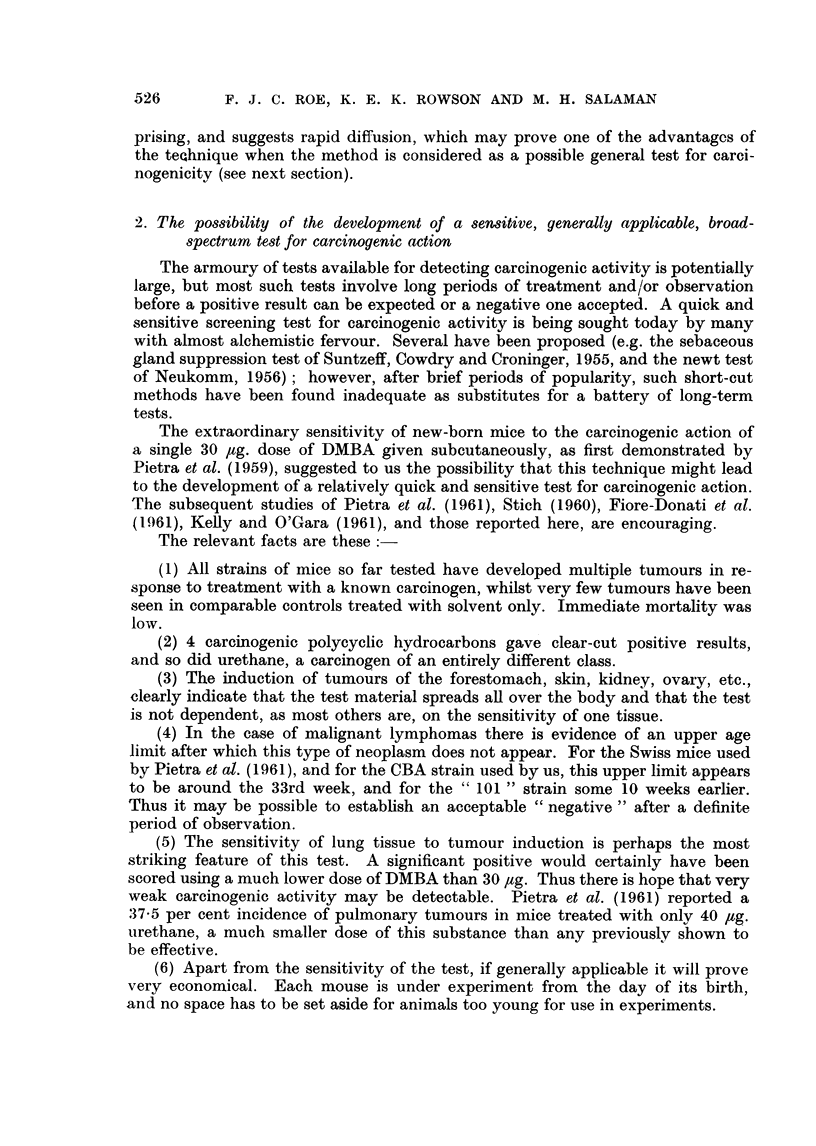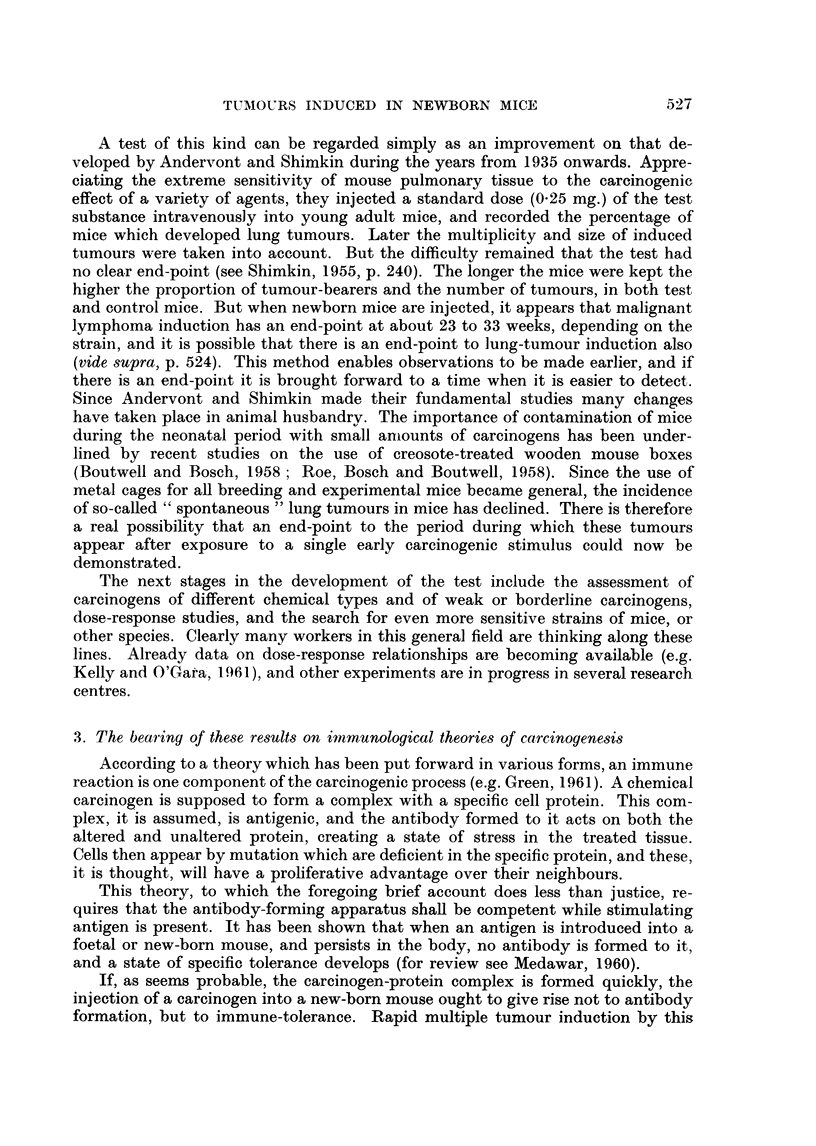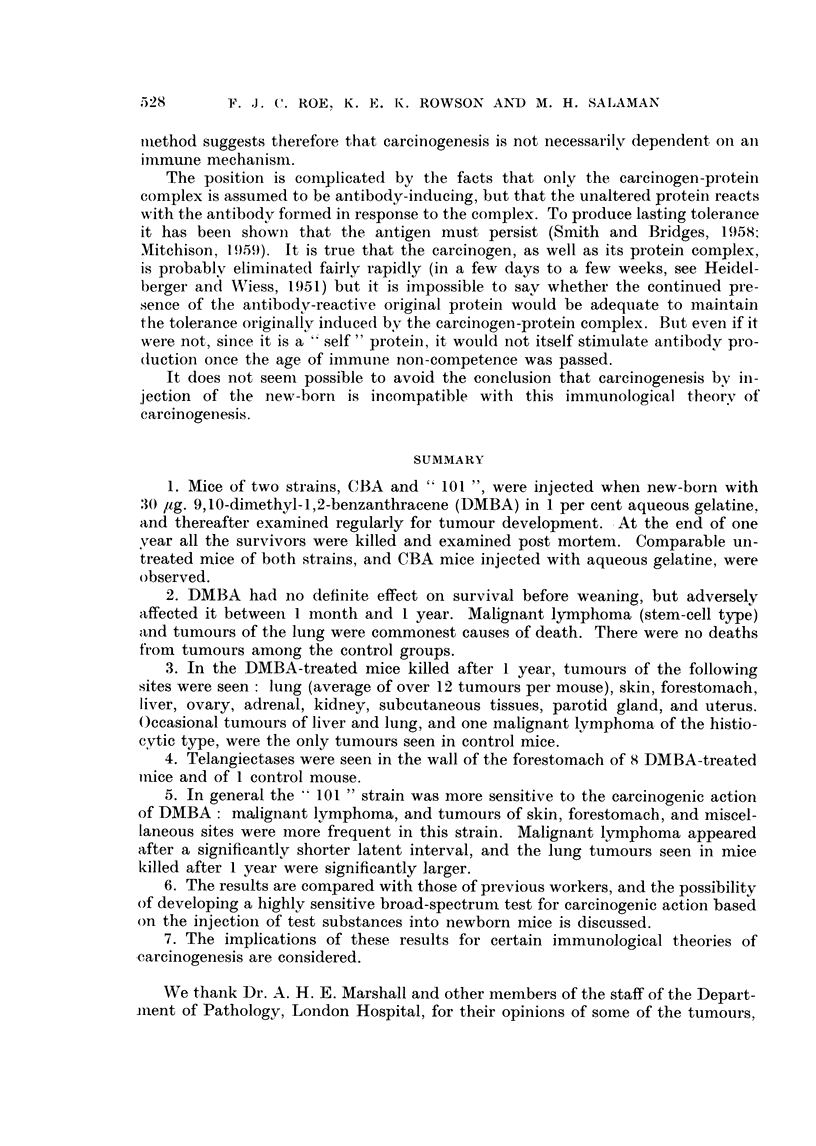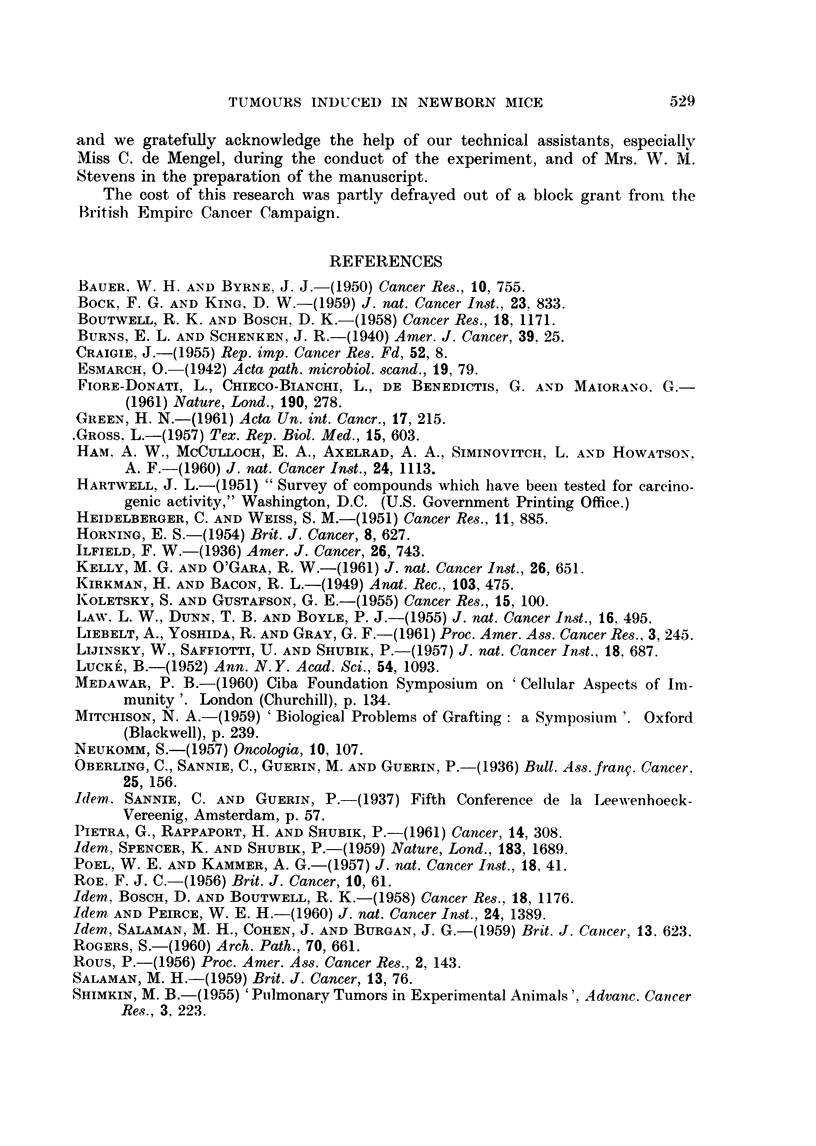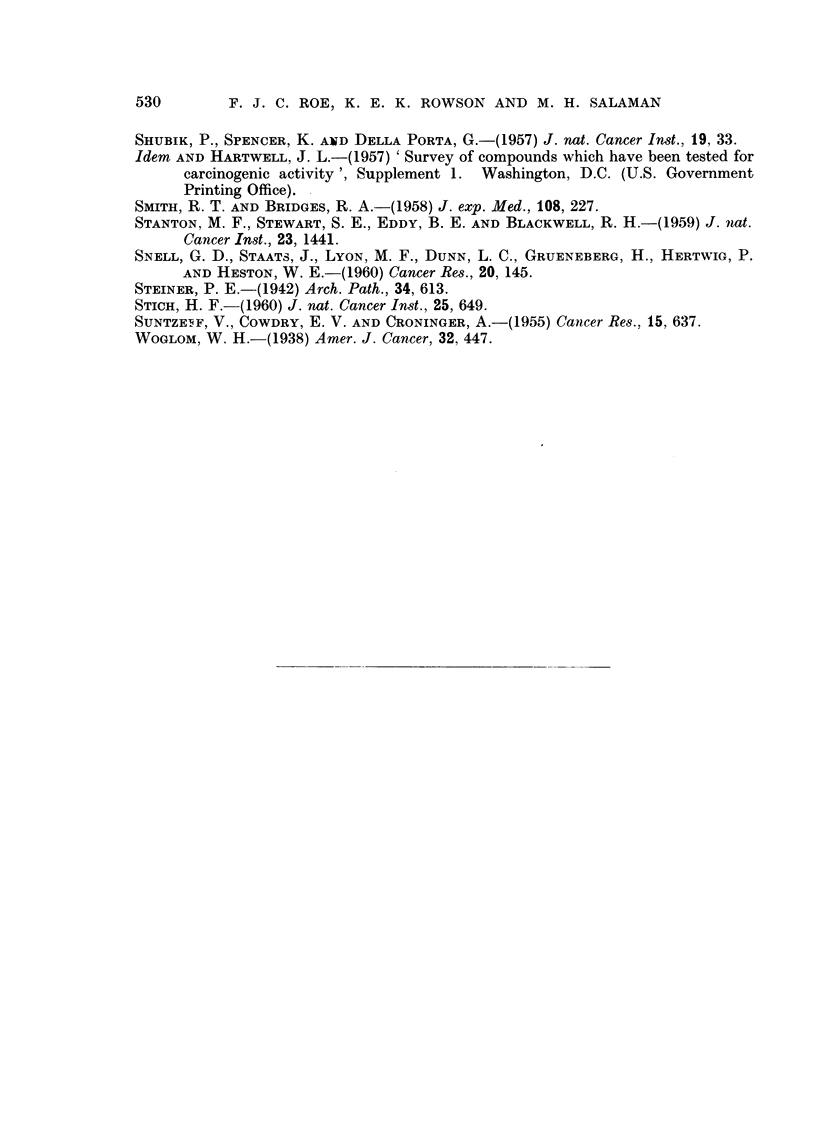# Tumours of Many Sites Induced by Injection of Chemical Carcinogens into Newborn Mice, A Sensitive Test for Carcinogenesis: The Implications for Certain Immunological Theories

**DOI:** 10.1038/bjc.1961.62

**Published:** 1961-09

**Authors:** F. J. C. Roe, K. E. K. Rowson, M. H. Salaman

## Abstract

**Images:**


### TUMOURS OF MANY SITES INDUCED BY INJECTION OF CHEMICAL CARCINOGENS INTO NEWBORN MICE. A SENSITIVE TEST FOR CARCINOGENESIS: THE IMPLICATIONS FOR CERTAIN IMMUNOLOGICAL THEORIES.

F. J. C. ROE*, K. E. K. ROWSON AND M. H. SALAMAN

*From the Cancer Research Department, London Hospital Medical College, Turner Street, London, E.1*

Received for publication June 27, 1961

PIETRA, SPENCER AND SHUBIK (1959) reported the induction of malignant lymphoma (lymphocytic leukaemia) and lung adenomas in Swiss mice injected subcutaneously when new-born (less than 24-hour old), with 30 $\mu$g. 9,10-dimethyl-1,2-benzanthracene (DMBA) suspended in 1 per cent aqueous gelatine. Later, Stich (1960) obtained a similar result using a 60 $\mu$g. dose of DMBA.

We were interested in the report of Pietra *et al.* (1959) for two reasons. Firstly we were anxious to discover whether cell-free filtrates prepared from malignant lymphoma induced by the injection of a carcinogen could themselves be passed to other mice by means of cell-free filtrates (e.g. Gross, 1957), and this method of chemical induction of lymphoma seemed very suitable. Secondly, the method described by Pietra *et al.* (1959) offered the possibility of a highly sensitive test for carcinogenic activity. In the latter connection it was important to find out whether other strains of mice would respond in the same way as the Swiss mice used by Pietra *et al.*, and also whether leukaemia and lung tumours could be induced by the injection of other carcinogens, both strong and weak, into new-born mice.

At the time that the present paper was ready for submission a second paper by Pietra and his colleagues (Pietra, Rappaport and Shubik, 1961) was published. The latter extended their earlier findings in four directions: firstly they confirmed their original observation with DMBA; secondly they tested 4 other carcinogens, 3,4-benzopyrene, 20-methylcholanthrene, 1,2:5,6-dibenzanthracene, and urethane by the same method; thirdly they compared the subcutaneous and intraperitoneal routes of administration for each of the 5 carcinogens: and fourthly they reported the induction of tumours at sites other than the lung and the lymphatic system. Even more recently Fiore-Donati *et al.* (1961) have recorded similar findings in an experiment in which new-born mice were injected with urethane: and Kelly and O'Gara (1961) have obtained similar results with 20-methylcholanthrene and 1,2:5,6-dibenzanthracene.

Work reported in the present paper confirms and extends the studies of Pietra and his colleagues. Two inbred strains of mice were used, and their response to DMBA compared. Induction of tumours of many sites, including those mentioned by Pietra *et al.* (1961), is described. The testing of cell-free filtrates prepared from

* Present address: Chester Beatty Research Institute, Institute of Cancer Research: Royal Cancer Hospital, Fulham Road, London, S.W.3.

carcinogen-induced malignant lymphoma will be the subject of a later communication.

### MATERIAL AND METHODS

*Mice.*—" CBA " and " 101 " inbred strains, both obtained originally from the MRC Radiobiological Research Unit, Harwell, were used (for further details see Snell *et al.*, 1960). Metal cages were used throughout the experiment. Diet 41B and water were provided *ad libitum.*

*Chemicals.*—9,10-dimethyl-1,2-benzanthracene (DMBA) was obtained from L. Light & Co. ; gelatine from British Drug Houses ; and croton oil from Stafford Allen and Co.

*Preparation and administration of DMBA/gelatine suspension.*—The methods of preparation and administration described by Pietra, Spencer, and Shubik (1959) were followed precisely. Thirty $\mu$g. DMBA suspended in 15 $\mu$l. of 1 per cent aqueous gelatine were injected subcutaneously in the interscapular region into test groups of both strains. One per cent aqueous gelatine alone was injected into a control group of the CBA strain. Untreated mice of both strains were also kept as controls. The general layout of the experiment is shown in Table I.

Litters which averaged 5–6 mice each were allotted randomly to test and control series, until groups of the required size were formed (7 to 11 litters per group —see Table I).

All mice were less than 24 hours old when injected.

*Subsequent conduct of the experiments.*—Litters were housed separately until weaning, at which time the mice were numbered on the ears and re-housed in boxes of 8 to 10 according to group and sex. A proportion of the mice failed to survive until weaning (Table I), but it was not possible to examine post mortem any of those which died.

After re-housing the mice were carefully examined, at first once weekly and, from the 10th week onwards, twice weekly until they were 1 year old, for evidence of malignant lymphoma, tumours of other sites, and other lesions. Sick mice were killed and examined post mortem, and at the end of the year all the survivors were killed and similarly examined.

Routine post mortem examination included a close scrutiny of the stomach wall after fixation by dilatation with formal saline.

### RESULTS

In both strains, groups injected with DMBA survived less well, and had a higher incidence of malignant lymphoma, lung tumours, skin tumours, tumours of the fore-stomach, and tumours of other sites, than uninjected mice or mice injected with suspending medium only. These differences are considered below under separate headings.

### *Survival*

It is doubtful whether treatment with DMBA had any unfavourable effect on survival during the first month of life (see Column 6 in Table I). The overall higher death rate during this period in CBAs as compared with " 101 "s, was probably due to the higher incidence of litter-eating in the former. This was common in our CBA colony at the time that the experiment was begun.

TABLE I.—*Effect of the Subcutaneous Injection of 30 µg. DMBA into New-born CBA and "101"-strain Mice : Survival and Incidence of Malignant Lymphomata*

| Group | Strain | Treatment | Number of litters | Mice alive at 1 day | Deaths during first month | Deaths between 1 month and 1 year | Causes of death between 1 month and 1 year: Malignant lymphoma | Other neo-plasms | Other causes | Not examined post mortem* | Mice alive at 1 year |
|---|---|---|---|---|---|---|---|---|---|---|---|
| 1 | CBA | DMBA in aqueous gelatine | 8 | 44 | 5† | 18 | 6 | 2 | 3 | 7 | 21 |
| 2 | ,, | Aqueous gelatine | 7 | 44 | 17† | 1 | 0 | 0 | 1 | 0 | 26 |
| 3 | ,, | None | 10 | 49 | 10† | 0 | 0 | 0 | 0 | 0 | 39 |
| 4 | 101 | DMBA in aqueous gelatine | 11 | 57 | 12 | 19 | 9 | 2 | 3 | 5 | 26 |
| 5 | ,, | None | 9 | 47 | 3 | 4 | 0 | 0 | 1 | 3 | 40 |
| | | | | 241 | 47 | 42 | | 27 | | 15 | 152 |

N.B.—

† The large numbers of deaths in CBA mice (Groups 1–3) during the first month is not a peculiar feature of the present experiment : eating of litters by mothers was common in our CBA colony at the time.

* Because of advanced decomposition.

Deaths between the end of the first month and one year were far more numerous in the DMBA-treated groups of both strains than in the control groups (for details see column 7 in Table I). The commonest causes of death during this period were malignant lymphoma of the stem-cell type (Pietra *et al.*, 1961) involving the thymus, and tumours of the lung. Six DMBA-treated CBAs, and 9 DMBA-treated "101"s died from lymphoma, and 2 mice of each strain from multiple, large, and/or malignant, lung tumours. Three DMBA-treated CBAs and 1 DMBA-treated "101" died without tumours of any site. No tumours were seen in either of 2 control mice (one from Group 2, and one from Group 5). No post mortem examination was possible in 15 mice (Table I). Combining the results from the two strains : 37/84 DMBA-treated mice died between 1 month and 1 year, whereas only 5/110 untreated or control-injected mice did so.

*Malignant lymphoma with thymic involvement*

The earliest clearcut difference between the DMBA-treated and the control groups was in the incidence of malignant lymphoma. As shown in Tables I and II a total of 15 cases were seen in the two groups treated with DMBA. Only one very late case was seen in the control groups. This was in an untreated "101"

TABLE II.—*Malignant Lymphoma Arising in Mice Injected when New-born with 30 μg. DMBA*

*DMBA-treated CBA mice (Group 1)*

| Litter | Mice alive at 1 month ♀ | ♂ | Number which developed malignant lymphoma ♀ | ♂ | Induction time (weeks) |
|---|---|---|---|---|---|
| A | 3 | 3 | 1 | — | 29 |
| B | 3 | 0 | — | — | |
| C | 4 | 0 | — | — | |
| D | 4 | 2 | 2 | — | 22, 22 |
| E | 6 | 0 | 1 | — | 32 |
| F | 1 | 4 | 1 | 1 | 29, 22 |
| G | 4 | 1 | — | — | |
| H | 3 | 1 | — | — | |
| | 28 | 11 | 5 | 1 | |
| | 39 | | 6 (15·4%) | | Av. = 26·0 weeks |

*DMBA-treated "101"-strain Mice (Group 4)*

| Litter | ♀ | ♂ | ♀ | ♂ | Induction time (weeks) |
|---|---|---|---|---|---|
| I | 1 | 3 | — | 2 | 10, 13 |
| J | 4 | 3 | — | 2 | 16, 20 |
| K | 0 | 5 | — | — | |
| L | 0 | 0 | — | — | |
| M | 0 | 2 | — | 2 | 13, 15 |
| N | 2 | 2 | — | — | |
| O | 1 | 3 | — | 1 | 25 |
| P | 2 | 4 | — | — | |
| Q | 5 | 3 | 2 | — | 13, 23 |
| R | 2 | 3 | — | — | |
| S | 0 | 0 | — | — | |
| | 17 | 28 | 2 | 7 | Av. = 16·5 weeks |
| | 45 | | 9 (20%) | | |

mouse, and was discovered at routine post mortem at one year. It was of a different histological type from the other cases (*vide infra*). Table II shows that the cases of malignant lymphoma were distributed randomly between the DMBA-treated litters. It is doubtful whether there was a real sex difference in incidence in either strain ; in fact, the apparent differences were in the opposite direction in the two strains. The total incidence was 20 per cent of " 101 " mice, and 15·4 per cent of CBA mice, alive at 1 month, but this slight difference was associated with a striking difference in average induction time ; 16·5 weeks in " 101 "s, and 26 weeks in CBAs. The latter difference was analysed by the " $t$ " test and found significant ($P < 0{\cdot}01$). This analysis, however, did not take into account the possibility that some of the DMBA-treated mice which died without post mortem may have had malignant lymphoma. This is unlikely, because they were not thought to have thymic tumours or enlarged lymph nodes when examined a few days before death, and because in both strains the average time of death of the

FIG. 1.—Comparison of induction-time of malignant lymphoma in CBA and " 101 "-strain mice after the injection of 30 μg. DMBA when newly born. This figure shows that deaths from unascertained causes occurred, for the most part, after the period during which malignant lymphoma appeared.

unexamined mice was considerably longer than that of the mice with lymphoma, and similar to that of mice dying without it (Fig. 1).

All the cases of malignant lymphoma which arose in the DMBA treated mice were of the histological type referred to by Pietra *et al.* (1961) as " stem cell ". In all of them there was gross enlargement of the thymus, so that it filled more than half the thoracic cavity, and an accompanying enlargement of some or all the lymph glands (axillary, inguinal, cervical, lumbar, pararenal, mediastinal, etc.). The spleen, on the other hand, was usually of normal size, and the liver always so.

The malignant lymphoma seen in the control untreated " 101 " mouse had a different distribution throughout the lymphatic system, and appeared quite different microscopically. There was slight enlargement of the left lobe of the thymus and more marked enlargement of the left pararenal lymph gland. The mesenteric lymph gland and spleen were also moderately enlarged, but the lymph glands in the neck, axillae, inguinal, and lumbar regions were of normal size. Microscopically the enlarged glands and thymus consisted predominantly of histiocytes with abundant eosinophilic cytoplasm. Mitotic figures were plentiful. The disease in this mouse was considered to be of the type referred to by Pietra

*et al.* (1961) as "histiocytic". No cases of lymphoma of the "lymphocytic" type, as described by Pietra and his colleagues, were seen.

*Lung tumours*

In both strains, mice treated with DMBA developed multiple benign, and occasional malignant, tumours of the lungs. By comparison, untreated mice of both strains and gelatine-injected CBAs developed a negligible number of such tumours, none of them malignant.

Pulmonary tumours were certainly present in several of the mice dying early from malignant lymphoma. However these were not counted because of the difficulty of distinguishing, except by microscopic examination, between adenomas and metastatic lymphomatous deposits. Apart from these, 4/10 DMBA-treated mice dying before they were 1 year old, had lung tumours.

TABLE III.—*Incidence of Lung Tumours 52 Weeks After Injection of* 30 $\mu$g. *DMBA into New-born CBA and "* 101 *"-strain Mice*

| Group | Strain | Treatment | Mice alive at 52 weeks* | Mice with lung tumours/mice examined | Total tumours | Average lung tumours per mouse examined | Average diameter of largest tumour (mm.) |
|---|---|---|---|---|---|---|---|
| 1 | CBA | DMBA/Gelatine | 21 | 16/18 | 222† | 12·2 | 1·8 |
| 2 | ,, | Gelatine | 26 | 0/25 | 0 | — | — |
| 3 | ,, | None | 39 | 3/39 | 6 | 0·16 | 1·2 |
| 4 | 101 | DMBA/Gelatine | 26 | 24/25 | 327† | 13·1 | 3·4 |
| 5 | ,, | None | 40 | 6‡/40 | 10‡ | 0·25 | 1·3 |

* Three mice from Group 1, and one each from Groups 2 and 4 died during the 52nd week and were too decomposed when found for post mortem examination.

† One malignant adenocarcinoma.

‡ Five of these mice, bearing 9 of the 10 tumours come from two litters and were housed together from weaning till death. It is possible that contamination with a carcinogen was responsible.

Table III shows the incidence of lung tumours in mice which survived for one year and were then killed. The DMBA-treated mice of both strains had an average of 12–13 tumours per mouse, whereas the incidence in the control groups was less than 0·3 tumours per mouse. The strains appeared to differ in their response to DMBA in that the average size of the largest tumours in the " 101 " mice was almost double that of the largest tumour in CBA mice. This difference was analysed by the "*t*" test and found to be significant ($P < 0{\cdot}01$). In fact not only the largest, but all the lung tumours in " 101 " mice tended to be larger than those in CBAs, and there can be no doubt that if records of the sizes of all tumours were available for comparison the difference would be even more significant.

Histologically all the tumours were of the well-known adenomatous type. Two of the tumours, one in a CBA mouse and one in a " 101 ", were adherent to the chest wall and had metastased to the mediastinum and upper surface of the diaphragm. Apart from these undoubtedly malignant tumours there were others which showed microscopic evidence of infiltration of surrounding pulmonary tissue.

There was no sex difference in the lung tumour response of " 101 "-strain mice to treatment with DMBA : 16 males which survived the year had an average of

13·2 tumours per mouse, against an average of 12·8 in 9 females. No comparison was possible in the case of CBAs since only 2 males of Group 1 came to post mortem at 52 weeks.

*Tumours of the skin*

Benign papillomas and sebaceous adenomas of the skin were seen in 1 out of 18 CBA and in 7 out of 25 " 101 " DMBA-treated mice which were examined post mortem at 52 weeks. All arose between the 35th and 52nd weeks, and the majority were on the head and face. One of the sebaceous adenomas is shown in Fig. 2. No skin tumours were seen in 104 control mice examined at the same time. No malignant skin tumours were seen in any group.

*Tumours and telangiectasia of the forestomach*

Close examination of the stomach was not at first included in the routine post mortem examination. However, when the first batch of mice from Group 4 (DMBA-treated " 101 "s) were killed and examined, papillomas and bright red patches were seen in the wall of the forestomach. Thereafter all stomachs were carefully examined, after distension with fixative. Only tumours of 1 mm. diameter and over were counted, and special care was taken in interpreting irregular projections in the vicinity of the ridge between forestomach and glandular stomach. The results are shown in Table IV.

TABLE IV.—*Papillomas and Telangiectases in the Forestomachs of 52-week old CBA and " 101 "-strain Mice Injected when New-born with 30 µg. DMBA*

| Group | Strain | Treatment | Number of mice examined for lesions of fore-stomach | Mice with papillomas of fore-stomach | Total papillomas | Papillomas 2 mm. diameter or more | Mice with telangiec-tases | Total telangiec-tases |
|---|---|---|---|---|---|---|---|---|
| 1 | CBA | DMBA/Gelatine | 22 | 2 | 2 | 1 | 2 | 5 |
| 2 | ,, | Gelatine | 23 | 0 | 0 | 0 | 0 | 0 |
| 3 | ,, | None | 38 | 0 | 0 | 0 | 1 | 1 |
| 4 | 101 | DMBA/Gelatine | 20 | 16 | 64 | 7 | 6 | 15 |
| 5 | ,, | None | 30 | 0 | 0 | 0 | 0 | 0 |

Papillomas were seen in DMBA-treated mice of both strains but not in any of the control groups. The incidence was far higher in the " 101 " than in the CBA strain. Most of the papillomas were about 1 mm. diameter but a few exceeded 2 mm. diameter (Fig. 3). There were no malignant tumours.

The occurrence of bright red patches in the wall of the forestomach has not to our knowledge been reported previously. They were seen in both strains (Table IV). The majority were circular and some were as large as 2·5 mm. diameter. Six of these lesions were examined microscopically : they were found to consist of groups of dilated capillaries, in fact typical *telangiectases* (Fig. 4). In several of the mice which had these lesions, and in some that did not, there were macroscopically similar lesions in the proximity of one or more of the Peyer's patches of the small intestine. Unfortunately no precise records were made of the latter in the present experiment. However, vascular lesions of the gut wall are not uncommon in mice treated with DMBA or urethane as young adults (unpublished data). Microscopic examination of the latter has shown them to be either telangiectases, or haemorrhages possibly derived from pre-existing telangiectases.

*Hepatic tumours*

Three mice in Group 1, 2 in Group 3, and 1 in each of the other 3 groups had parenchymal-cell liver tumours. Clearly this difference in incidence between the DMBA-treated and control groups was minimal. However it was interesting that the three CBA mice of group 1 had multiple (more than 10) tumours each, whereas the 3 control CBAs from Groups 2 and 3 had 2, 1 and 1 tumours respectively. Similarly, the mouse in Group 4 with liver tumours had 4 of them, whereas the mouse from Group 5 had only 1.

Histologically, several of the tumours showed cytoplasmic inclusions similar to those described by Burns and Schenken (1940) and Craigie (1955). In view of the sex difference observed by these authors in $C_3H$ mice, it is of interest that 7 out of the 8 mice reported here were males. The remaining mouse, a gelatine-injected control of Group 2, was a virgin female, exactly 1 year old when killed. The tumour in this mouse was 5 mm. in diameter and of typical gross appearance : it was not examined histologically.

*Tumours of other sites*

Eleven mice, all of which had been treated with DMBA, had tumours of other sites. These are listed in Table V, and four of them are illustrated in Fig. 5–8.

Since only one or two examples of each kind of tumour were seen it is not proposed to describe them in detail.

*Papillonephritis*

Naked eye evidence of this disease, consisting of swelling, pallor, or calcification of the renal papilla, and cicatrisation of the renal cortex, was observed in 9 out of 26 DMBA-treated " 101 "-strain mice, and in 20 out of 39 untreated control mice of the same strain, killed when 1 year old. No cases of the disease were seen in CBA mice.

EXPLANATION OF PLATES

Lesions seen in mice injected when less than 24 hours old with 30 μg. DMBA in 1 per cent aqueous gelatine, and killed when 52 weeks old. Stained H. and E.

FIG. 2.—Sebaceous adenoma from skin of abdominal wall of a " 101 " ♂. ×48.

FIG. 3.—Papilloma of wall of forestomach of a CBA ♀. ×35.

FIG. 4.—Telangiectasis in wall of forestomach of a CBA ♀. A group of grossly dilated capillaries are seen beneath the keratinized squamous epithelium. The wide gap between the dilated vessels and the muscle layers is an artifact. ×70.

FIG. 5.—Granulosa-cell tumour of ovary from a CBA. ×370.

FIG. 6.—Cortical adnoma in the kidney of a " 101 " ♂. ×65.

FIG. 7.—Malignant haemangioma infiltrating subcutaneous and muscle layers of thigh and abdominal wall of " 101 " ♀. Remnants of voluntary muscle are seen. Parts of this tumour were more cellular and mitotically active. ×330.

FIG. 8.—Tumour from parotid gland of a " 101 " ♂. Opinion was divided as to the nature of this tumour. The absence of cross-striations in a P.T.A. stain failed to support a diagnosis of rhabdomyosarcoma and the high mitotic rate and arrangement of P.T.A.-positive material in the cytoplasm, as bunches of thin threads, did not fit the diagnosis of oxyphilic granular cell adenoma (onkocytoma). The final diagnosis lay between the latter and leiomyosarcoma. ×370.

2

3

4

5

6

7

8

Roe, Rowson and Salaman.

TABLE V.—*Miscellaneous Tumours seen in 52-week old CBA and " 101 "-strain Mice, Injected when New-born with 30 μg. DMBA (Excluding Malignant Lymphoma and Tumours of Lung, Skin, Stomach, and Liver)*

| Group | Strain | Treatment | Mice examined at 52 weeks | Miscellaneous tumours | Figure Number |
|---|---|---|---|---|---|
| 1 | CBA | DMBA/Gelatine | 18 | Carcinoma of adrenal cortex (1 case) | 5 |
| | | | | Granulosa-cell tumour of ovary (2 cases) | |
| 2 | ,, | Gelatine | 25 | None | — |
| 3 | ,, | None | 39 | None | — |
| 4 | 101 | DMBA/Gelatine | 25 | Cortical adenoma of kidney (2 cases, one with 2 adenomas) | 6 |
| | | | | Fibrosarcoma in subcutaneous tissues of ear (1 case) | |
| | | | | Haemangioma (3 cases, one in subcutaneous tissues of neck, one in uterus, and one highly malignant tumour involving thigh and body wall) | 7 |
| | | | | Myeloid leukaemia (1 case) | |
| | | | | ? Leiomyosarcoma of parotid gland (1 case) | 8 |
| 5 | ,, | None | 40 | None | — |

The occurrence of papillonephritis in " 101 "-strain mice has been commented upon previously (Roe *et al.*, 1959). The present observation confirms our previous impression that treatment with DMBA does not materially affect the incidence of the disease (Roe and Peirce, 1960).

### DISCUSSION

### 1. *Significance of experimental results*

In general our experimental findings confirmed those of Pietra *et al.* (1959, 1961), Stich (1960), Fiore-Donati *et al.* (1961), and Kelly and O'Gara (1961), but several new observations were made.

Injection of 30 μg. DMBA subcutaneously into new-born mice had no pronounced effect on survival up to the age of weaning, but adversely affected it to a significant degree between the ages of 1 month and 1 year. During the latter period malignant lymphoma (stem-cell type) and multiple lung tumours accounted for about half the deaths, but there was also an increased death-rate from causes other than neoplasia. Cases of malignant lymphoma were seen in the DMBA-treated groups of both strains of mice : all cases arose between the 10th and 32nd weeks, a finding closely similar to that in the earlier studies referred to above. The incidence was similar in the two strains (20 per cent in " 101 "s and 15·4 per cent in CBAs), but the time of appearance was significantly earlier in the " 101 " strain. There was no evidence that susceptibility differed between litters of the same strain.

Multiple pulmonary tumours were seen in DMBA-treated mice of both strains. Most of these were apparently benign, but two were manifestly malignant. At one year the average number of pulmonary tumours per survivor was similar in the two strains, but there was a significant strain difference in the average size of the largest adenoma in each mouse. The fact that all lung tumours (not only the largest) tended to be larger in the " 101 " strain could be explained by earlier appearance in this strain, as in the case of the malignant lymphomas. However, a recent observation by Rogers (1960) throws doubt on this view : he found that equal-sized pieces of small and large lung tumours, when transplanted to the ears of genetically compatible host mice, grew at different rates, the latter growing faster than the former. This would suggest that in the present experiment the lung tumours in the " 101 " strain had a higher growth-rate than those in the CBA strain. Further experiment is required to settle this point.

The results as a whole indicated that the CBA strain is less responsive to the carcinogenic action of DMBA than the " 101 " strain. It was therefore surprising that the final incidence of lung tumours was so similar. Of course the tumour counts refer only to macroscopically visible surface adenomas, and the incidence in the two strains might have appeared quite different if smaller and more deeply situated lesions had been taken into account. Further, the comparison was made after the arbitrary period of one year. If this period had been shorter or longer a difference in tumour incidence might have been seen. The period of onset of malignant lymphomas was complete at the 32nd week. We do not know whether the appearance of lung tumours was confined to a definite period, or continued throughout life ; and, unfortunately, the very recently reported experiments of Kelly and O'Gara (1961) were not carried on long enough to answer this question. In some ways the data on size and incidence of tumours presented here suggests that the former is true. This is a fascinating problem which does not seem to have been adequately studied (see review on pulmonary tumours in experimental animals by Shimkin, 1955).

In our experiments there was no definite correlation between treatment with DMBA and incidence of hepatomas, although it was curious that in DMBA-treated mice they were usually multiple, while in controls two was the most seen in any one mouse. In the experiments of Pietra and his colleagues occasional hepatomas were seen in carcinogen-treated mice, but none in controls. More recently, in a preliminary report, Liebelt, Yoshida, and Gray (1961) describe a striking enhancement of the incidence of hepatomata in $C_3$Hf mice injected with urethane when less than 24 hours old but not when 6–8 weeks old. The presence of cytoplasmic inclusions, similar to those described by Burns and Schenken (1940) and Craigie (1955), in the hepatomas of both DMBA-treated and control mice in the present experiment suggests that DMBA was not the only causative agent. It may be that the administration of carcinogen to new-born mice enhances the effect of another agent.

The induction of skin tumours by administration of DMBA to suckling mice was perhaps not surprising, in view of certain recent observations. Rous (1956) observed skin tumours in untreated mice, and attributed them to the fact that the mice had been bred in wooden cages preserved with creosote. Poel and Kammer (1957), Lijinsky, Saffiotti, and Shubik (1957), Shubik, Spencer, and Della Porta (1957) and Boutwell and Bosch (1958) conducted experiments which confirmed that relatively slight exposure of infant or adult mice to creosote led to the induction or initiation of skin tumours. The fact that most of the tumours seen in the present experiment arose on the face and head fits in with a previous observation of one of us (Roe, 1956) that the skin tumours which arise 36 weeks or more after the application of 300 $\mu$g. DMBA to the dorsal skin of 8-week old mice are also predominantly on the head and face.

Papillomas of the forestomach were not noted by Pietra and his colleagues. However such tumours would be overlooked if routine post mortem examination did not include dilatation of the stomach with fixative and examination of the lining when fixed. The discovery of papillomas in this site was not altogether surprising, in view of the recent demonstration by Bock and King (1959) of the relatively high sensitivity of the forestomach epithelium to tumour induction.

The telangiectasia of the stomach wall is also a new observation. Dilatation of vessels and haemorrhages in the wall of the small gut, particularly in the proximity of Peyer's patches, in mice treated as adults with DMBA or urethane have been seen by us in previous experiments, but we do not recall seeing similar lesions in the forestomach. Unfortunately no record of the frequency of intestinal telangiectases and haemorrhages was made in the present experiment, though it is known that they occurred. There was no tendency for papillomas and telangiectases of the forestomach to occur at the same site.

Several other miscellaneous tumours, in particular granulosa-cell tumours of the ovary, and haemangiomata of the various sites, occurred both in the present experiment and in those of Pietra *et al.* (1961). There can be little doubt that they resulted from carcinogen treatment. The induction of a carcinoma of the adrenal cortex, of an unusual tumour of the parotid gland (? leiomyosarcoma) (Fig. 8), and of several adenomata of the renal cortex, by a chemical carcinogen are observations which may have importance in their own right.

Search through the literature listed by Hartwell (1951) and Shubik and Hartwell (1957) revealed no examples of induction of any of these tumours by carcinogenic polycyclic hydrocarbons, except the induction of renal tumours by the introduction of massive doses directly into the kidney (Ilfield, 1936 ; Oberling, Sannie and Guerin, 1937 ; Esmarch, 1942). Even this drastic technique sometimes yielded negative results (e.g. Oberling *et al.*, 1936 ; Woglom, 1938). On the other hand, adenomata of the renal cortex have been repeatedly induced by other agents, e.g. by polyoma virus in mice (Stanton *et al.*, 1959) and hamsters (Ham *et al.*, 1960), by another virus in frogs (Lucké, 1952), by oestrogens in male hamsters (Kirkman and Bacon, 1949 ; Horning, 1954), and by X-irradiation in rats (Koletsky and Gustafson, 1955).

Steiner (1942) introduced carcinogenic polycyclic hydrocarbons into the salivary glands of mice, rats, guinea-pigs, and rabbits. In all species except the rabbit he observed a variety of tumours including squamous carcinomas, acantho-adenocarcinomas, adenocarcinomas, spindle-cell sarcomas, and cavernous haemangiosarcomas. However he did not see any tumours similar in microscopic appearance to the one in the present experiment (Fig. 8), and no such tumours are recorded in experiments prior to 1942 (for reference see Steiner, 1942). It is possible that some of the parotid gland tumours seen by Bauer and Byrne (1950) were in fact of this histological type. Like Steiner they induced their tumours by the introduction of the carcinogen (20-methylcholanthrene) directly into the salivary gland.

The parotid tumours induced by the polyoma virus (Law, Dunn and Boyle, 1955 ; Salaman, 1959) are histologically quite different from the tumour seen in the present experiment.

The absence of tumours at the site of injection of DMBA is somewhat surprising, and suggests rapid diffusion, which may prove one of the advantages of the technique when the method is considered as a possible general test for carcinogenicity (see next section).

2. *The possibility of the development of a sensitive, generally applicable, broad-spectrum test for carcinogenic action*

The armoury of tests available for detecting carcinogenic activity is potentially large, but most such tests involve long periods of treatment and/or observation before a positive result can be expected or a negative one accepted. A quick and sensitive screening test for carcinogenic activity is being sought today by many with almost alchemistic fervour. Several have been proposed (e.g. the sebaceous gland suppression test of Suntzeff, Cowdry and Croninger, 1955, and the newt test of Neukomm, 1956) ; however, after brief periods of popularity, such short-cut methods have been found inadequate as substitutes for a battery of long-term tests.

The extraordinary sensitivity of new-born mice to the carcinogenic action of a single 30 $\mu$g. dose of DMBA given subcutaneously, as first demonstrated by Pietra *et al.* (1959), suggested to us the possibility that this technique might lead to the development of a relatively quick and sensitive test for carcinogenic action. The subsequent studies of Pietra *et al.* (1961), Stich (1960), Fiore-Donati *et al.* (1961), Kelly and O'Gara (1961), and those reported here, are encouraging.

The relevant facts are these :—

(1) All strains of mice so far tested have developed multiple tumours in response to treatment with a known carcinogen, whilst very few tumours have been seen in comparable controls treated with solvent only. Immediate mortality was low.

(2) 4 carcinogenic polycyclic hydrocarbons gave clear-cut positive results, and so did urethane, a carcinogen of an entirely different class.

(3) The induction of tumours of the forestomach, skin, kidney, ovary, etc., clearly indicate that the test material spreads all over the body and that the test is not dependent, as most others are, on the sensitivity of one tissue.

(4) In the case of malignant lymphomas there is evidence of an upper age limit after which this type of neoplasm does not appear. For the Swiss mice used by Pietra *et al.* (1961), and for the CBA strain used by us, this upper limit appears to be around the 33rd week, and for the " 101 " strain some 10 weeks earlier. Thus it may be possible to establish an acceptable " negative " after a definite period of observation.

(5) The sensitivity of lung tissue to tumour induction is perhaps the most striking feature of this test. A significant positive would certainly have been scored using a much lower dose of DMBA than 30 $\mu$g. Thus there is hope that very weak carcinogenic activity may be detectable. Pietra *et al.* (1961) reported a 37·5 per cent incidence of pulmonary tumours in mice treated with only 40 $\mu$g. urethane, a much smaller dose of this substance than any previously shown to be effective.

(6) Apart from the sensitivity of the test, if generally applicable it will prove very economical. Each mouse is under experiment from the day of its birth, and no space has to be set aside for animals too young for use in experiments.

A test of this kind can be regarded simply as an improvement on that developed by Andervont and Shimkin during the years from 1935 onwards. Appreciating the extreme sensitivity of mouse pulmonary tissue to the carcinogenic effect of a variety of agents, they injected a standard dose (0·25 mg.) of the test substance intravenously into young adult mice, and recorded the percentage of mice which developed lung tumours. Later the multiplicity and size of induced tumours were taken into account. But the difficulty remained that the test had no clear end-point (see Shimkin, 1955, p. 240). The longer the mice were kept the higher the proportion of tumour-bearers and the number of tumours, in both test and control mice. But when newborn mice are injected, it appears that malignant lymphoma induction has an end-point at about 23 to 33 weeks, depending on the strain, and it is possible that there is an end-point to lung-tumour induction also (*vide supra*, p. 524). This method enables observations to be made earlier, and if there is an end-point it is brought forward to a time when it is easier to detect. Since Andervont and Shimkin made their fundamental studies many changes have taken place in animal husbandry. The importance of contamination of mice during the neonatal period with small amounts of carcinogens has been underlined by recent studies on the use of creosote-treated wooden mouse boxes (Boutwell and Bosch, 1958 ; Roe, Bosch and Boutwell, 1958). Since the use of metal cages for all breeding and experimental mice became general, the incidence of so-called " spontaneous " lung tumours in mice has declined. There is therefore a real possibility that an end-point to the period during which these tumours appear after exposure to a single early carcinogenic stimulus could now be demonstrated.

The next stages in the development of the test include the assessment of carcinogens of different chemical types and of weak or borderline carcinogens, dose-response studies, and the search for even more sensitive strains of mice, or other species. Clearly many workers in this general field are thinking along these lines. Already data on dose-response relationships are becoming available (e.g. Kelly and O'Gara, 1961), and other experiments are in progress in several research centres.

3. *The bearing of these results on immunological theories of carcinogenesis*

According to a theory which has been put forward in various forms, an immune reaction is one component of the carcinogenic process (e.g. Green, 1961). A chemical carcinogen is supposed to form a complex with a specific cell protein. This complex, it is assumed, is antigenic, and the antibody formed to it acts on both the altered and unaltered protein, creating a state of stress in the treated tissue. Cells then appear by mutation which are deficient in the specific protein, and these, it is thought, will have a proliferative advantage over their neighbours.

This theory, to which the foregoing brief account does less than justice, requires that the antibody-forming apparatus shall be competent while stimulating antigen is present. It has been shown that when an antigen is introduced into a foetal or new-born mouse, and persists in the body, no antibody is formed to it, and a state of specific tolerance develops (for review see Medawar, 1960).

If, as seems probable, the carcinogen-protein complex is formed quickly, the injection of a carcinogen into a new-born mouse ought to give rise not to antibody formation, but to immune-tolerance. Rapid multiple tumour induction by this method suggests therefore that carcinogenesis is not necessarily dependent on an immune mechanism.

The position is complicated by the facts that only the carcinogen-protein complex is assumed to be antibody-inducing, but that the unaltered protein reacts with the antibody formed in response to the complex. To produce lasting tolerance it has been shown that the antigen must persist (Smith and Bridges, 1958; Mitchison, 1959). It is true that the carcinogen, as well as its protein complex, is probably eliminated fairly rapidly (in a few days to a few weeks, see Heidelberger and Wiess, 1951) but it is impossible to say whether the continued presence of the antibody-reactive original protein would be adequate to maintain the tolerance originally induced by the carcinogen-protein complex. But even if it were not, since it is a " self " protein, it would not itself stimulate antibody production once the age of immune non-competence was passed.

It does not seem possible to avoid the conclusion that carcinogenesis by injection of the new-born is incompatible with this immunological theory of carcinogenesis.

### SUMMARY

1. Mice of two strains, CBA and " 101 ", were injected when new-born with 30 $\mu$g. 9,10-dimethyl-1,2-benzanthracene (DMBA) in 1 per cent aqueous gelatine, and thereafter examined regularly for tumour development. At the end of one year all the survivors were killed and examined post mortem. Comparable untreated mice of both strains, and CBA mice injected with aqueous gelatine, were observed.

2. DMBA had no definite effect on survival before weaning, but adversely affected it between 1 month and 1 year. Malignant lymphoma (stem-cell type) and tumours of the lung were commonest causes of death. There were no deaths from tumours among the control groups.

3. In the DMBA-treated mice killed after 1 year, tumours of the following sites were seen : lung (average of over 12 tumours per mouse), skin, forestomach, liver, ovary, adrenal, kidney, subcutaneous tissues, parotid gland, and uterus. Occasional tumours of liver and lung, and one malignant lymphoma of the histiocytic type, were the only tumours seen in control mice.

4. Telangiectases were seen in the wall of the forestomach of 8 DMBA-treated mice and of 1 control mouse.

5. In general the " 101 " strain was more sensitive to the carcinogenic action of DMBA : malignant lymphoma, and tumours of skin, forestomach, and miscellaneous sites were more frequent in this strain. Malignant lymphoma appeared after a significantly shorter latent interval, and the lung tumours seen in mice killed after 1 year were significantly larger.

6. The results are compared with those of previous workers, and the possibility of developing a highly sensitive broad-spectrum test for carcinogenic action based on the injection of test substances into newborn mice is discussed.

7. The implications of these results for certain immunological theories of carcinogenesis are considered.

We thank Dr. A. H. E. Marshall and other members of the staff of the Department of Pathology, London Hospital, for their opinions of some of the tumours, and we gratefully acknowledge the help of our technical assistants, especially Miss C. de Mengel, during the conduct of the experiment, and of Mrs. W. M. Stevens in the preparation of the manuscript.

The cost of this research was partly defrayed out of a block grant from the British Empire Cancer Campaign.

### REFERENCES

BAUER, W. H. AND BYRNE, J. J.—(1950) *Cancer Res.*, **10**, 755.
BOCK, F. G. AND KING, D. W.—(1959) *J. nat. Cancer Inst.*, **23**, 833.
BOUTWELL, R. K. AND BOSCH, D. K.—(1958) *Cancer Res.*, **18**, 1171.
BURNS, E. L. AND SCHENKEN, J. R.—(1940) *Amer. J. Cancer*, **39**, 25.
CRAIGIE, J.—(1955) *Rep. imp. Cancer Res. Fd*, **52**, 8.
ESMARCH, O.—(1942) *Acta path. microbiol. scand.*, **19**, 79.
FIORE-DONATI, L., CHIECO-BIANCHI, L., DE BENEDICTIS, G. AND MAIORANO, G.—(1961) *Nature, Lond.*, **190**, 278.
GREEN, H. N.—(1961) *Acta Un. int. Cancr.*, **17**, 215.
.GROSS, L.—(1957) *Tex. Rep. Biol. Med.*, **15**, 603.
HAM, A. W., MCCULLOCH, E. A., AXELRAD, A. A., SIMINOVITCH, L. AND HOWATSON, A. F.—(1960) *J. nat. Cancer Inst.*, **24**, 1113.
HARTWELL, J. L.—(1951) " Survey of compounds which have been tested for carcinogenic activity," Washington, D.C. (U.S. Government Printing Office.)
HEIDELBERGER, C. AND WEISS, S. M.—(1951) *Cancer Res.*, **11**, 885.
HORNING, E. S.—(1954) *Brit. J. Cancer*, **8**, 627.
ILFIELD, F. W.—(1936) *Amer. J. Cancer*, **26**, 743.
KELLY, M. G. AND O'GARA, R. W.—(1961) *J. nat. Cancer Inst.*, **26**, 651.
KIRKMAN, H. AND BACON, R. L.—(1949) *Anat. Rec.*, **103**, 475.
KOLETSKY, S. AND GUSTAFSON, G. E.—(1955) *Cancer Res.*, **15**, 100.
LAW, L. W., DUNN, T. B. AND BOYLE, P. J.—(1955) *J. nat. Cancer Inst.*, **16**, 495.
LIEBELT, A., YOSHIDA, R. AND GRAY, G. F.—(1961) *Proc. Amer. Ass. Cancer Res.*, **3**, 245.
LIJINSKY, W., SAFFIOTTI, U. AND SHUBIK, P.—(1957) *J. nat. Cancer Inst.*, **18**, 687.
LUCKÉ, B.—(1952) *Ann. N.Y. Acad. Sci.*, **54**, 1093.
MEDAWAR, P. B.—(1960) Ciba Foundation Symposium on ' Cellular Aspects of Immunity '. London (Churchill), p. 134.
MITCHISON, N. A.—(1959) ' Biological Problems of Grafting : a Symposium '. Oxford (Blackwell), p. 239.
NEUKOMM, S.—(1957) *Oncologia*, **10**, 107.
OBERLING, C., SANNIE, C., GUERIN, M. AND GUERIN, P.—(1936) *Bull. Ass. franç. Cancer*, **25**, 156.
*Idem*, SANNIE, C. AND GUERIN, P.—(1937) Fifth Conference de la Leewenhoeck-Vereenig, Amsterdam, p. 57.
PIETRA, G., RAPPAPORT, H. AND SHUBIK, P.—(1961) *Cancer*, **14**, 308.
*Idem*, SPENCER, K. AND SHUBIK, P.—(1959) *Nature, Lond.*, **183**, 1689.
POEL, W. E. AND KAMMER, A. G.—(1957) *J. nat. Cancer Inst.*, **18**, 41.
ROE, F. J. C.—(1956) *Brit. J. Cancer*, **10**, 61.
*Idem*, BOSCH, D. AND BOUTWELL, R. K.—(1958) *Cancer Res.*, **18**, 1176.
*Idem* AND PEIRCE, W. E. H.—(1960) *J. nat. Cancer Inst.*, **24**, 1389.
*Idem*, SALAMAN, M. H., COHEN, J. AND BURGAN, J. G.—(1959) *Brit. J. Cancer*, **13**, 623.
ROGERS, S.—(1960) *Arch. Path.*, **70**, 661.
ROUS, P.—(1956) *Proc. Amer. Ass. Cancer Res.*, **2**, 143.
SALAMAN, M. H.—(1959) *Brit. J. Cancer*, **13**, 76.
SHIMKIN, M. B.—(1955) ' Pulmonary Tumors in Experimental Animals ', *Advanc. Cancer Res.*, **3**, 223.

SHUBIK, P., SPENCER, K. AND DELLA PORTA, G.—(1957) *J. nat. Cancer Inst.*, **19**, 33.
*Idem* AND HARTWELL, J. L.—(1957) ' Survey of compounds which have been tested for carcinogenic activity ', Supplement 1. Washington, D.C. (U.S. Government Printing Office).
SMITH, R. T. AND BRIDGES, R. A.—(1958) *J. exp. Med.*, **108**, 227.
STANTON, M. F., STEWART, S. E., EDDY, B. E. AND BLACKWELL, R. H.—(1959) *J. nat. Cancer Inst.*, **23**, 1441.
SNELL, G. D., STAATS, J., LYON, M. F., DUNN, L. C., GRUENEBERG, H., HERTWIG, P. AND HESTON, W. E.—(1960) *Cancer Res.*, **20**, 145.
STEINER, P. E.—(1942) *Arch. Path.*, **34**, 613.
STICH, H. F.—(1960) *J. nat. Cancer Inst.*, **25**, 649.
SUNTZEFF, V., COWDRY, E. V. AND CRONINGER, A.—(1955) *Cancer Res.*, **15**, 637.
WOGLOM, W. H.—(1938) *Amer. J. Cancer*, **32**, 447.